# The development and validation of the Palestinian children's traumatic events checklist in a war-torn environment

**DOI:** 10.1186/s12888-024-05731-1

**Published:** 2024-04-03

**Authors:** Guido Veronese, Fayez Mahamid, Dana Bdier, Hania Obaid, Federica Cavazzoni

**Affiliations:** 1 Department of Human Sciences and Education “R.Massa”, University of Milano-Bicocca, Milan, Italy; 2 Department of Counseling and Psychology, An-Najah National University, Nablus, Palestine; 3 Department of Psychology, Stellenbosch University, Stellenbosch, South Africa

**Keywords:** War children trauma, Military violence-exposure, Psychometric properties, Palestine

## Abstract

Traumatic experiences in childhood can lead to trauma symptoms and impaired mental health, especially when children are exposed to war and political violence. Despite significant attention to child's exposure to traumas, few instruments to detect potentially traumatic events have been validated psychometrically. Our study aimed to develop, adapt and validate a user-friendly traumatic events checklist in Palestinian children living in three areas affected by low-intensity war and ongoing political and military violence. 965 Palestinian children (494 males and 471 females) living in the Gaza Strip, West Bank and East Jerusalem were administered with a tailor-made Traumatic Events checklist, Children Impact of Events scale, and Strengths and Difficulties Scale. Exploratory and Confirmatory factor analysis was run to detect the factorial structure of the checklist. Furthermore, ANOVA was performed to identify statistically significant demographic differences among participants. A three factors structure emerged with Political violence-related traumatic experiences (PVTE), military violence against individuals (MVI), and military violence against individuals and families (MVF). Gaza children and adolescents resulted in being the most exposed to potentially traumatic events. The instrument can clearly portray potentially traumatic experiences in children exposed to violent events and adverse childhood experiences.

## Introduction

Children have been the unseen victims of armed conflicts throughout history. Today, wars and hostilities spread to many places worldwide [1]. According to UNICEF [2], war negatively impacts children's mental health, affecting their adjustment, self-concept, resilience, and quality of life.

The Israeli–Palestinian conflict could be regarded as continuing rather than a single, highly disrupting incident. Many of the violent war-like events children face in Palestine extend indefinitely. In this context, many generations of children have grown up knowing nothing except violent war-like events, conflicts, and traumatizing conditions [3].

The effects of war and violence on the mental health of children and adults are well known. They are often expressed through severe and chronic reactive psychological syndromes, including post-traumatic stress disorder (PTSD), anxiety, and behavioral problems [4]. The potentially traumatizing circumstances that can affect children when exposed to war-torn environments are less known. The Palestinian population living in the occupied territories has been subjected to continuous violence, such as shooting, bombardment, and physical injuries. As a result of this ongoing crisis, Palestinians, especially children, have developed severe psychological traumas [2].

Several studies reported that Palestinian children suffer from traumatic symptoms due to ongoing political conflict between the Israeli army and Palestinians. A survey conducted by Espié et al. [5] found that among 1254 Palestinian children living in Gaza Strip, 23.2% reported post-traumatic stress disorder (PTSD), 17.3% anxiety disorder (other than PTSD or acute stress disorder), and 15.3% depression. PTSD was more frequently identified in children ≤ 15 years old. Accordingly, among children ≤ 15 years old, episodes significantly associated with PTSD included witnessing a murder or physical abuse, receiving threats, and property destruction or loss. According to the meta-analysis study completed by Agbaria et al. [6], 28 articles, representing 32 samples with 15,121 participants from Gaza Strip and West Bank, were included in the study to measure the prevalence of post-traumatic stress disorder among Palestinian children and adolescents exposed to political violence. The survey showed the prevalence of PTSD was 36% (95% CI 30–41%; Sub-group analysis showed that the PTSD prevalence did not differ according to the West Bank and Gaza Strip regions.

Understanding how children experience such traumatic events as war, violence, and abuse requires measures and procedures to detect these potentially traumatic events (PTEs) children experience in their daily lives [7]. Since 1987, when the diagnosis of PTSD was extended to children and adolescents, efforts to study children's exposure and reactions to possible stressors have used various methods. Further, we acknowledge that not always adverse and extreme experiences will necessarily lead to trauma symptoms and syndromes. As a result, it is difficult to characterize the prevalence of PTEs or to accurately assess them among children without using standardized and validated instruments [8].

From a relativist's perspective, where the notion holds that all human behaviors are culturally patterned, the cross-cultural validity of trauma experience has been questioned for many years. The categorization of trauma experiences is based on agreed-upon notions of how a person is supposed to interpret adversities. These notions, and therefore experiences of trauma, are shaped by culturally informed protective strategies, environmental constraints, and context-specific forms of violence. These context-driven influences on the experiences of trauma pose several challenges in addressing traumatic events, understanding and assessing differing sociocultural contexts, therefore developing sensitive cultural methods to classify PTEs [9, 10].

Several studies have been implemented to validate trauma screening checklists in international contexts. Viola et al. [11] tested the validity of the Childhood Trauma Questionnaire in a multicounty study (CTQ). The CTQ is a self-administered questionnaire that persons aged 12 years and older can fill out. Five elements–including emotional abuse, physical abuse, sexual abuse, emotional neglect, and physical neglect–were used to evaluate each subtype of childhood maltreatment. Europe and Asia had the lowest CTQ figures compared to South America, with the highest estimates. Specifically, studies from China, the Netherlands and the United Kingdom yielded the most conservative maltreatment estimates.

Choi et al. [12] developed and validated the Traumatic Events Screening Inventory (TESI) for vulnerable children in Chicago. The TESI advances beyond preceding instrumentation by encompassing both a child's and parent's version of the tool to assess a wide range of adverse childhood experiences. The questionnaire was given to a group of consecutively seen intakes from child psychiatric outpatient clinics. Identifying if the child has experienced a traumatic event provides the clinician with a broader insight into their functioning, aiding in treatment. The TESI has potential applications in clinical research and can be combined with other methods. More recent results showed strong validity and reliability indicators of TESI in assessing traumatic experiences among vulnerable children.

The Stressful Life Events Schedule for children and adolescents [13] encompasses over 300 PTEs clustered in severe and non-severe events. The Life Events and Difficulties Schedule (LEDS), which is an investigator-based semi-structured interview to measure stressful life events in children [14], represent a significant step forward in evaluating environmental stressors. Still, their use is limited because of the labor and time they involve. For example, specific tools for detecting PTEs have been developed by the UK police for children and their parents [15].

Hence, it is imperative for professionals dealing with kids and families who are war-torn and displaced to be sensible of and evaluate the numerous traumatic experiences kids have been confronted with [16].

In Palestine, over the years, numerous tools aimed at recording PTEs related to war and violence have been developed [4, 17]. For example, typical PTEs occurred among children during the first intifada were summarized in 10 items that were expanded in updated versions of the first tool.

The most advanced instrument detecting traumatic events related to war was construed by Thabet and Vostanis [18]. The original version of the Gaza Traumatic Checklist – Parent Form was constructed by the research branch of the Gaza Community Mental Health Programme and incorporated 17 items that canvassed various kinds of traumatic situations that the child may have encountered [19]. Considering the transformation of the political dispute in the territory, this checklist was adjusted to include new items, such as witnessing the bombardment of homes by helicopters, heavy artillery and tanks and witnessing mutilated bodies on television. Fathers and mothers were canvassed regarding the episodes their offspring had undergone in the past 12 months. The checklist scores were evaluated by summing the scores and scores stratification (low traumatic exposure for scores under 5, moderate exposure for scores between 5–9, and high exposure for scores of 10 and more). The authors also evaluated the impact of specified traumatic events, i.e. the presence or absence of each event, i.e. the presence or absence of each checklist item. More recently, El-Khodary et al. [20] readapted the War-Traumatic Events Checklist (W-TECh) with Gaza children*.* The authors were the ones to construct the W-TECh. Some items were derived from Thabet et al. [21] prior study. The items were adapted to suit the most recent armed conflict consequences in the Gaza Strip. The W-TECh is formed of 28 binary answers separated into three clusters. The first comprised experiencing personal trauma when kids and teenagers are exposed to direct war-related trauma, such as being struck or harmed by bullets. Witnessing traumatic events with children or adolescents as observers (e.g., family members, friends, or neighbors) being shot and/or injured during the war. Finally, a third dimension is seeing the demolition of a property when victims witnessed demolishing their home, school, and/or farm during the war. None of these instruments in Palestine was validated and tested for their psychometric properties.

Hence, the existing trauma instruments are still limited in detecting clusters of war-related trauma events. There is a need for a user-friendly and psychometrically robust tool to assess traumatic events among Palestinian children living amidst chaotic and catastrophic conditions that would help to explain how children might develop specific traumatic symptoms and diagnoses of trauma. The current study aims to develop and validate a new trauma checklist and test its psychometric properties and factorial structure as an easy-to-use and robust assessment instrument for Palestine children affected by war and political violence.

## Methodology

### Participants

We conducted our study with 965 Palestinian children: 494 males and 471 females. The majority, 36.8% of participants, were from cities, 34.8% were from villages, 27.3% were from Palestinian internally displaced camps, and 9% were from the Bedouin community. Regarding geographical areas, 60.5% of participants were from West Bank, 9.7% from Jerusalem, and 29.7% from the Gaza Strip. 36.3% were aged 8–10, 36.2% were aged 11–12, and 26.5% were aged 13–14. To be included in the study, participants must be 1) Palestinians, 2) not been previously diagnosed with mental health disorders, and 3) Native Arabic speakers. The study was approved by An-Najah Institutional Review Board (IRB) before data collection was administered.

#### Measures

*Children Trauma Checklist*: The Palestinian trauma checklist (TCL) is a self-report measure comprising 48 items designed to test traumatic symptoms among Palestinian children. We developed the scale based on previous trauma-related literature and psychometric instruments designed to test traumatic experiences [5, 9, 19]. The initial TCL consisted of 53 items, Five items were deleted due to low factor loadings, the scale ended up with 48 items comprising three subscales: (1) Political violence-related traumatic experiences (PVTE), including the following items (2, 3, 4, 5, 22, 23, 24, 31, 33, 34, 35, 36, 37, 38, 39, 40, 41, 42, 43, 45, 46 and 47); (2) Military violence against individuals (MVI), including the following items (1, 7, 8, 9, 11, 12, 13, 14, 15, 19, 20, and 21); (3) Military violence against individuals and family (MVIF), including the following items (6, 10, 16, 17, 18, 25, 26, 27, 28, 29, 30, 32, 44, and 48).

The checklist has a two-point scale (Yes = 1 and No = 0).

Ten Palestinian professional experts in psychology, counseling, and social work reviewed the items of the scale for content validity and comprehensiveness; 80% as a percentage of agreement between experts was used for each item. The researchers modified certain items based on the advice offered by the committee members.

*Children Revised Impact of Event Scale-Arabic Version* (CRIES-13A), [22, 23]: The scale is a version of the Impact of Event Scale by Dyregrov et al. [24] designed and adapted to measure the psychological trauma of children exposed to traumatic events. The original scale has three dimensions: intrusion, avoidance, and hyperarousal. Items are graded on a four-point Likert scale. Sample items included: "Do you think about erasing the event that shocked you from your memory?", "Do you have difficulty concentrating?" and "Do you try to avoid thinking about the shocking event?". The CRIES-13A revealed a high level of internal consistency in this sample of Palestinian children (α = 0.88).

*Strengths and Difficulties scale-Arabic version* (SDQ): The SDQ is a brief behavioral assessment questionnaire that contains 25 characteristics, some favorable and others unfavorable. The SDQ seeks to measure psychological and social issues and assets (e.g. pro-social behaviors) in children and young people aged 3–16 via a multi-informant approach. Parents and teachers can detect difficulties and strengths among 3- to 16-year-olds, whereas 11- to 16-year-olds can articulate their challenges and strengths. The questionnaire comprises 25 items apportioned evenly between five scales assessing emotional symptoms, conduct issues, hyperactivity-inattention, peer problems, and pro-social behavior. Excluding the pro-social scale, the combined scale score reflects the difficulties, illustrating the intensity and the matter of psychosocial suffering. The pro-social scale indicates a child's pro-social characteristics [22]. The SDQ revealed a high level of internal consistency in this sample of Palestinian children (α = 0.91).

#### Data analysis

We used Expletory Factor Analysis (EFA) to discover the factor structure of the measure among 450 participants of the total sample, while confirmatory factor analysis (CFA) using AMOS 29 software was implemented among 515 participants to test the dimensionality of the scale’s construct. The model yielded satisfactory indications concerning the goodness of fit, showing CFI = 0.97, GFI = 0.98, NFI = 0.95, RFI = 0.96, IFI = 0.97 and TLI = 0.96. Descriptive statistics were used to assess the characteristics of the TCL scale in the Palestinian Context. Moreover, the Analysis of Variance test (ANOVA) was performed to evaluate the differences in TCL among participants through demographic variables; gender, region, residence and age. Concurrent validity was found for the TCL scale by testing the correlation between SDQ, CRIES-13A, and the TCL scale. Finally, Guttmann Split-Half, and Cronbach's Alpha, were calculated to assess the scale's internal consistency and test–retest reliability.

#### Procedures

Our study was conducted in March 2023. Written parental consent was requested and obtained before starting the research. Participants and their families were carefully informed about the study’s purposes. Children were free to respond partially and withdraw from the survey at any moment if distressed by any protocol questions. All the data were anonymous and analyzed in an aggregate mode. Participation in the study was voluntary, and no rewards were offered. Local trained social workers distributed the study tools to students, they also clarified items to respondents. Questionnaires were collected in the classrooms during school hours. The accomplishment of the research protocol lasted about 30 min. The arranged settings offered a safe and relational space where pupils could freely express their perceptions and opinions. Of the 980 participants recruited, 965 questionnaires were retained and analyzed. Fifteen questionnaires were excluded from the analysis because of the responses’ inaccuracy. The research protocol adhered to the APA’s ethical principles. Finally, the Institutional Review Boards (IRB) of a An-Najah National University approved the research before the data collection was initiated.

## Findings

**Fig. 1:**
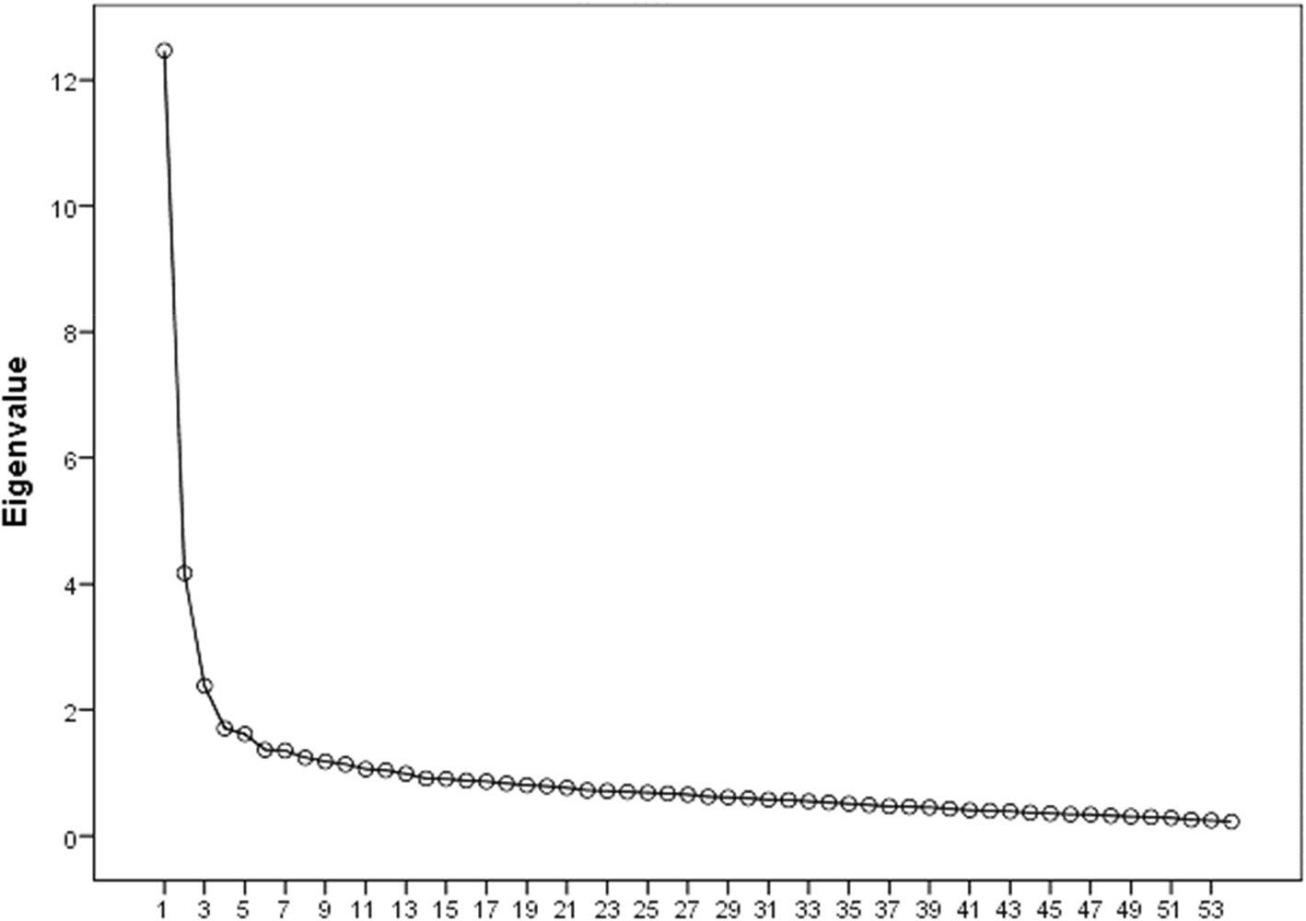
Number of factors based on eigenvalue

**Fig. 2:**
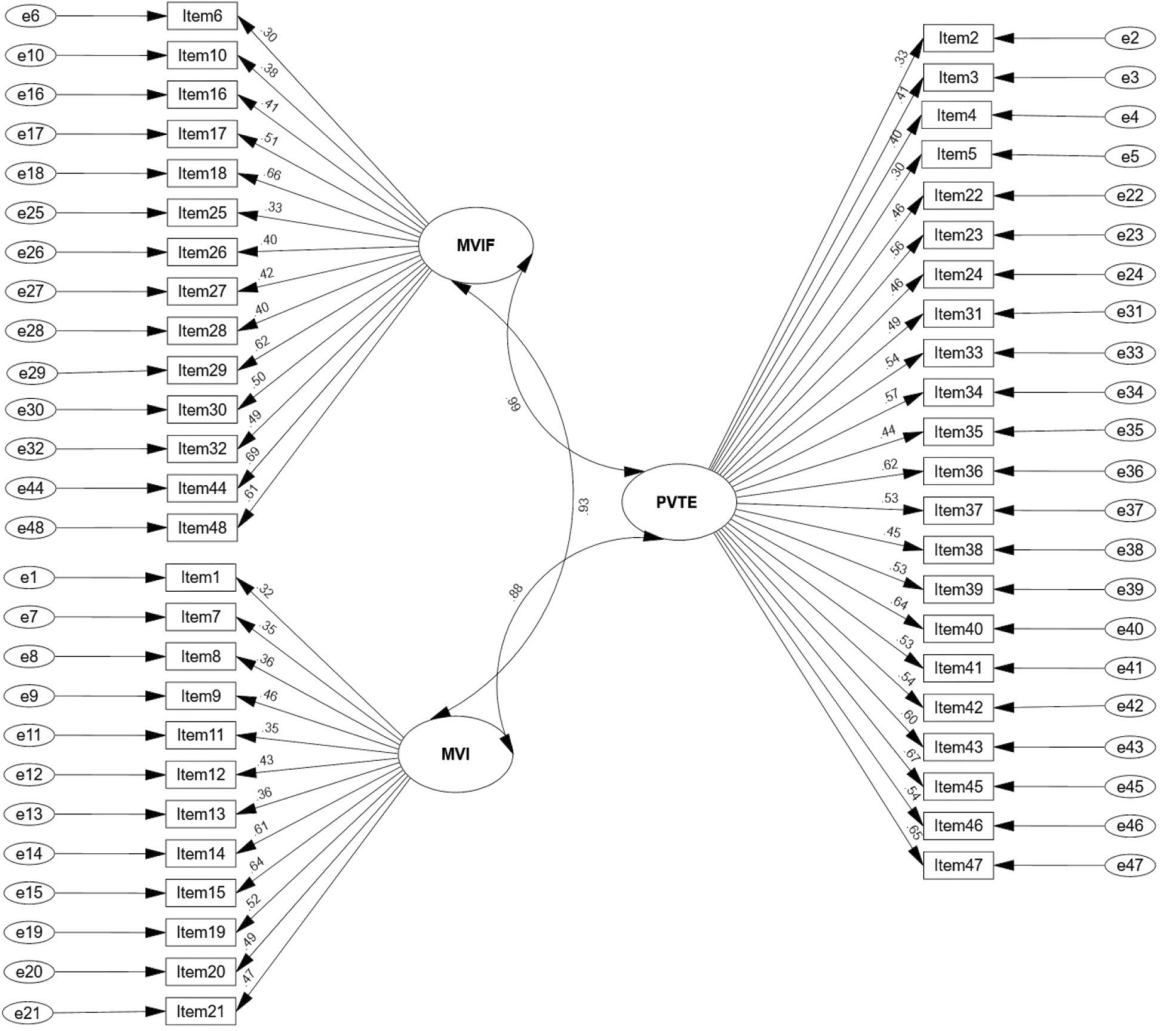
Confirmatory factor analysis of TCL within the Palestinian context. *Political violence-related traumatic experiences (PVTE); Military violence against individuals (MVI); Military violence against individuals and family (MVIF)*

We conducted EFA (see Fig. 1 and Table 1), and CFA (see Fig. 2) for TCL, with results of three principal constructs yielded from the analysis: (1) Political violence-related traumatic experiences (PVTE), (2) violence against individuals (MVI), and (3) military against individuals and family (MVF). The initial model assumed that TCL is multidimensional and comprised of a three-factor structure. The model fitting indicators produced χ2 = 2492.1, DF = 79 and *P* ≤ 0.001, indicating the model was unfit. As the model lacked acceptable fit indicators, modified indicators were used to determine whether covariance should be allowed among item errors. Byrne (2016) suggested using an adjusted index with residuals between two items (± 2.58). Table 1 shows the covariance of the three-scale construct.

**Table 1:** Covariance of the three TCL constructs

Construct	Estimate	SE	CR	*P*
PVTE	.85	.08	9.23	***.001
MVI	.91	.09	8.47	***.001
MVF	.87	.06	9.54	***.001

^***^P significance ≤ *0.001*

Incremental fit indices were also calculated for a stepwise addition of error covariance where a value closest to one indicates the best model fit. The resulting incremental indices values (CFI = 0.97, GFI = 0.98, NFI = 0.95, RFI = 0.96, IFI = 0.97, and TLI = 0.96) were all ≥ 0.95, indicating very good model fit. The RMSEA value was.041, less than the cut-off value of close to 0.06. Finally, item loading values ranged from 0.50 to 0.75, within the acceptable range of high loading values (all > 0.50).

### Concurrent validity

A Pearson Correlation Coefficient was calculated between the total scores of CRIES, SDQ difficulties, and TCL scales to evaluate the concurrent validity of the TCL scale. The results of concurrent validity are presented in Table 2.

**Table 2:** Pearson Correlation between TCL, SDQ difficulties, and CRIES scales (*n* = 965)

Scale	*1*	*2*	*3*	*4*	*5*	*6*	*7*	*8*	*9*
*1.Avoidance*	1	.61^a^	.63^a^	.19^a^	.17^a^	.43^a^	.35^a^	.24^a^	.30^a^
*2.Intrusion*		1	.62^a^	.19^a^	.22^a^	.44^a^	.19^a^	.23^a^	.28^a^
*3.Hyperarousal*			1	.16^a^	.17^a^	.41^a^	.18^a^	.25^a^	.31^a^
*4.PVTE*				1	.73^a^	.49^a^	.41^a^	.15^a^	.23^a^
*5.MVI*					1	.39^a^	.33^a^	.19^a^	.18^a^
*6.MVIF*						1	.34^a^	.21^a^	.28^a^
*7.TCL total*							1	.42^a^	.39^a^
*8.CRIES*								1	.17^a^
*9.SDQ* difficulties									1

^a^Correlation is significant at the 0.01 level (2-tailed)

The findings of correlational analysis showed that avoidance positively correlated with intrusion (*r* = 0.61; *p* < *0.01*), hyperarousal (*r* = 0.63; *p* < *0.01*), PVTE (*r* = 0.19; *p* < *0.01*), MVI (*r* = 0.17; *p* < *0.01*), MVIF (*r* = 0.43; *p* < *0.01*), total score of TCL (*r* = 0.35; *p* < *0.01*), CRIES(*r* = 0.35; *p* < *0.01*), and SDQ difficulties (*r* = 0.30; *p* < *0.01*). Additionally, intrusion positively correlated with hyperarousal (*r* = 0.62; *p* < 0.01), PVTE (*r* = 0.19; *p* < *0.01*), MVI (*r* = 0.22; *p* < *0.01*), MVIF (*r* = 0.44; *p* < *0.01*), total score of TCL(*r* = 0.19; *p* < *0.01*), CRIES(*r* = 0.23; *p* < *0.01*), and SDQ difficulties (*r* = 0.28; *p* < *0.01*). Besides, hyperarousal positively correlated with PVTE (*r* = 0.16; *p* < *0.01*), MVI (*r* = 0.17; *p* < *0.01*), MVIF (*r* = 0.41; *p* < *0.01*), total score of TCL(*r* = 0.18; *p* < *0.01*), CRIES(*r* = 0.25; *p* < *0.01*), and SDQ difficulties (*r* = 0.31; *p* < *0.01*). Moreover, PVTE positively correlated with MVI (*r* = 0.73; *p* < *0.01*), MVIF (*r* = 0.49; *p* < *0.01*), total score of TCL(*r* = 0.41; *p* < *0.01*), CRIES(*r* = 0.15; *p* < *0.01*), and SDQ difficulties (*r* = 0.23; *p* < *0.01*). On the other hand, MVI positively correlated with MVIF (*r* = 0.39; *p* < *0.01*), total score of TCL(*r* = 0.33; *p* < *0.01*), CRIES(*r* = 0.19; *p* < *0.01*), and SDQ difficulties (*r* = 0.18; *p* < *0.01*). While, MVIF positively correlated with TCL(*r* = 0.34; *p* < *0.01*), CRIES(*r* = 0.21; *p* < *0.01*), and SDQ difficulties (*r* = 0.28; *p* < *0.01*). TCL total score positively correlated with CRIES(*r* = 0.42; *p* < *0.01*), and SDQ difficulties (*r* = 0.39; *p* < *0.01*). Finally, CRIES positively correlated with SDQ difficulties (*r* = 0.17; *p* < *0.01*).

### Reliability of the TCL scale

To test the reliability of the TCL scale, test–retest, Cronbach's Alpha, and Guttmann Split-Half were calculated, as shown in Table 3.

**Table 3:** Reliability analysis of TCL scale (*n* = 965)

Item	Items	Cronbach's Alpha if Item Deleted	Corrected Item-Total Correlation	Test–retest	Guttmann Split- Half	α
1	Did you inhale teargas?	.87	.55	.84		
2	Did you or any of your family members witness a night raid/day raid?	.86	.59	.85		
3	Did you witness painful hand tying during the moments of arrest?	.81	.58	.87		
4	Have you witnessed sniffer dogs during the moments of arrest?	.84	.63	.89		
5	Did you witness the demolition of homes/your school/of a friend/ relative?	.77	.64	.77		
6	Is your home threatened by demolition?	.85	.61	.82		
7	Have you been assaulted by settlers?	.84	.45	.84		
8	Have you been assaulted by soldiers?	.83	.56	.83		
9	Have you been assaulted by pigs or dogs owned by settlers?	.84	.66	.79		
10	Did you experience any attack against your school or your home?	.88	.61	.83		
11	Have you been injured during the participation in the great march of return?	.83	.74	.82		
12	Have you been detained by the Israeli army while going to school?	.87	.56	.79		
13	Were you subjected to gunshots?	.80	.47	.81		
14	Were you insulted on a military barricade?	.77	.67	.83		
15	Were you arrested?	.84	.52	.87		
16	Was your house subjected to shelling by tanks?	.90	.62	.88		
17	Was your house subjected to shelling by planes or drones?	.78	.69	.81		
18	Were you kept inside your house with your family by military forces?	.83	.44	.86		
19	Were you burnt by grenades or phosphorous bombs?	.84	.71	.84		
20	Have you ever been surrounded by shelling?	.86	.52	.79		
21	Were you injured or hurt during the war?	.87	.61	.78		
22	Did you have any fears of death as a result of the continuous shelling?	.81	.71	.86		
23	Were you deprived from medical care at the time that you needed it?	.83	.73	.81		
24	Were your deprived of water, food and electricity?	.84	.67	.86		
25	Were you and your family subjected to eviction?	.87	.65	.82		
26	Was one of your family members injured?	.79	.63	.84		
27	Was one of your family members arrested?	.87	.47	.81		
28	Did you see one of your family members beaten by the Israeli army?	.89	.53	.82		
29	Did you see one of your family members isolated by the Israeli army?	.83	.72	.83		
30	Did you lose a family member due to military violence?	.80	.62	.81		
31	Were you obliged to leave your house during the shelling?	.86	.61	.82		
32	Were you and your family forced to leave before the shelling or after the shelling?	.87	.55	.84		
33	Did you see a friend get killed?	.82	.62	.82		
34	Did you see a friend or friends get injured?	.89	.75	.81		
35	Did you see strangers get killed?	.77	.64	.87		
36	Did you see strangers injured?	.86	.58	.82		
37	Did you see a shooting?	.85	.62	.83		
38	Did you see the remains of a car that had been shelled by a plane?	.86	.71	.84		
39	Did you see the remains of a car that had been shelled by a tank?	.81	.67	.85		
40	Did you see funerals of martyrs?	.82	.68	.87		
41	Did you see body parts of martyrs or dead people?	.90	.72	.81		
42	Did you see people dying during the shelling?	.91	.54	.83		
43	Was your house demolished by the occupation while you are outside of it?	.82	.61	.84		
44	Was your land subjected to bulldozing by the occupation?	.87	.57	.83		
45	Was your area subjected to incursion?	.86	.63	.85		
46	Did you suffer any losses due to shelling?	.81	.62	.82		
47	Did you have losses in your house due to shelling?	.82	.64	.81		
48	Did your family lose its source of living as a result of military violence?	.87	.77	.83		
	***PVTE***			.86	.84	.92
	***MVI***			.84	.83	.90
	***MVIF***			.87	.82	.90
	***TCL total score***			.88	.86	.93

*TCL* Children trauma checklist, *PVTE* Political violence-related traumatic experiences, *MVI* Military violence against individuals, *MVIF* Military violence against individuals and family

Results of Cronbach's Alpha for internal consistency of the TCL scale showed a high level of reliability (α = 0.93). In addition, results of the split-half showed a high level of internal reliability (0.86). Test re-test of the trauma context was calculated by administering the scale to 200 participants from the original study sample three weeks after the first administration. The correlation between the first- and second-time trauma context checklist was 0.88, showing that the TCL scale is reliable in assessing traumatic symptoms in Palestinian children.

Differences in averages were noted in TCL due to several demographic variables; age, region, residence and gender as the following: Age (8–10, M = 0.59; 11–12, M = 0.58; 13–14, M = 0.62), region (West Bank, M = 0.57; Jerusalem, M = 0.59; Gaza, M = 0.65), Residence ( refugee camp, M = 0.61; Urban/city, M = 0.57; village, M = 0.61; Bedouin community, M = 0.70), and gender (Males, M = 0.60; Females, M = 0.59). To test the differences in TCL due to age, region, residence and gender, ANOVA test was conducted (see Table 4).

**Table 4:** Results of ANOVA test for differences in trauma exposure between study variables (*N* = 965)

Dependant	Source	SS	DF	MS	F	Sig
***TCL total***	**Age**	.412	2	.206	8.932	.000***
	**Region**	2.966	2	1.483	64.337	.000***
	**Residence**	.802	3	.267	11.599	.000***
	**Gender**	.002	1	.002	.089	.765
	**Error**	21.665	940	.023		
	**Corrected Total**	26.221	948			

***P significance ≤.001

Results of Table 4 showed significant differences in TCL due to age (*F* = 8.93, *P* ≤ 001), region (*F* = 64.33, *P* ≤ *001*), and residence (*F* = 11.59, *P* ≤ *001*). To compare the differences between categories of these variables, a Least Significant Difference (LSD) test was calculated (Table 5).

**Table 5:** LSD test to compare the difference in categories means (*N* = 965)

*TCL*				
***Age***	8–10	11–12	13–14	
8–10		.005	-.05*	
11–12			-.06*	
13–14				
***Region***	West bank	Jerusalem	Gaza	
West bank		-.04*	-.13*	
Jerusalem			-.08*	
Gaza				
***Residence***	Refugee camp	Urban/city	Village	Bedouin community
Refugee camp		.06*	.05	-.13*
Urban/city			-.05*	-.19*
Village				-.13*
Bedouin community				

*P significance ≤.05

Results of Table 5 showed significant differences in TCL due to age categories, children in the age group 13–14 reported more exposure to traumatic events compared with other age groups (MD = -0.05 &0.06*; P* ≤ *0.05*). Significant differences were also found in TCL due to region categories, children living in Gaza Strip reported more exposure to traumatic events compared with other children living in the West Bank and East Jerusalem (MD = -0.13 & -0.08; *P* ≤ *0.05*). Finally, significant differences were found in TCL due to place of residence, children living in Bedouin community reported more exposure to traumatic events compared with children living in refugee camps, cities and villages (MD = -0.13, -0.19, &—0.13; *P* ≤ *0.05*).

## Discussion

The current study was designed to develop and validate a context-specific trauma checklist to evaluate PTEs among Palestinian child victims of war and military violence. The findings of our study revealed a high concurrent validity of TCL in assessing traumatic events within the Palestinian context. A positive association was found between TCL, CRIES-13, and SDQ difficulties scales, which indicates that TCL is a valid and reliable method for assessing traumatic events following among Palestinian children. Results of CFA showed a stable construct of a three-factor structure of the TCL in assessing potentially traumatic events: (1) Political violence-related traumatic experiences (PVTE), (2) military violence against individuals (MVI), and (3) military violence against individuals and family (MVIF). Several previous studies indicated these components' importance in assessing children's traumatic experiences. Giraldo et al. [25] tested the psychometric properties of the trauma experiences scale for armed conflict contexts in Colombia. The scale ended up with two sub-scales: direct military and political violence experiences and indirect military and political violence experiences. Ibrahim et al. [26] conducted a study to determine the psychometric properties and diagnostic utility of the Posttraumatic Stress Disorder Checklist (PCL-5) as a screening instrument for war-affected displaced Kurdish and Arab populations. Results of CFA confirmed a stable construct of a four-factor solution of PCL-5 within the Kurdish people; military violence against individuals (during displacement), war-related event types (lifetime), event types experienced, and event types witnessed.

Political conflict is expected to increase the risk of trauma symptoms development among children, as our correlational analysis indicated. Overall, the majority of studies conducted in the Occupied Palestinian Territories have emphasized the high rates of dysfunction and maladaptation in Palestinian children and reported a high prevalence of trauma and common mental health problems and more severe disorders [19, 20]. Accordingly, our findings revealed pre-adolescents, adolescents, children living in extreme war-torn environments such as the Gaza Strip and in areas affected by high levels of political violence (Bedouin communities) as particularly exposed to PTEs and trauma symptoms.

Developing new tools to evaluate the exposure to PTEs will help mental healthcare providers to establish several therapeutic interventions that target Palestinian children particularly exposed to political and military violence, thus, trauma-related syndromes [27]. Our findings revealed an excellent validity of TCL in assessing the exposure to traumatic events, and statistically significant differences in exposure to PTEs due to age groups were observed. Children in the age group 13–14 years reported more exposure to traumatic events compared to age groups 8–10 and 10–12. One possible explanation for this result is that young children may be less directly exposed to violence and more protected in the domestic sphere. Our results align with previous findings, which showed that younger children had experienced fewer trauma events [8].

Our study results also showed significant differences in traumatic exposure due to regions, Palestinian children living in Gaza strip reported more exposure to traumatic events compared to children living in the West Bank and East Jerusalem. This result can be explained by the fact that Palestinian children living in Gaza experience more war-like episodes than elsewhere in the region, which can lead to traumatic stress reactions. The situation in the Gaza Strip is uncommon in the frequency with which children are exposed to war-related traumatic events daily and because of the long-term and ongoing nature of the conflict they experienced. Therefore, it is expected to see a high level of PTEs exposure among children living in Gaza compared with other children living in the West Bank and East Jerusalem. Similarly, El-Khodary et al. [20] tested traumatic experiences among 909 children living in Gaza Strip. The majority of children and adolescents experienced personal trauma (88.4%), witnessed trauma to others (83.7%) and observed demolition of property (88.3%) during the Gaza wars.

The results also indicated that children in Bedouin communities reported more PTEs than Palestinian children in urban and rural regions. This result can be explained as Palestinian children living in Bedouin communities are considered a part of unrecognized populations severely exposed to demolition, displacements and evictions from non-registered homes by the Israeli army. The Bedouins have been forcibly relocated several times to provide space for Israeli settlement growth, military firing zones and newly declared nature reserves. Massad et al. [28] examined exposures to traumatic events and mental health among 455 refugee children selected from 18 Bedouin communities; 44% of the participants in the study had probed psychiatric disorders. Exposure to traumatic events, fair/poor maternal self-rated mental health, and younger age were positively associated with child mental health problems.

### Limitations of the study

Our study has a few limitations that may offer opportunities for future studies; First, we collected our data using convenience and purposive sampling, which may limit our results' generalizability. Second, another limitation involves using the CRIES-13 and SDQ for investigating the convergent validity of the TCL. Both measures are restrained in investigating an ongoing traumatic reality, and the SDQ has never been validated within the Palestinian context. However, both scales are widely used and reliable instruments for investigating trauma in children that, in general, are scarce in the Palestinian mental health system. Finally, this research primarily focused on developing a context-specific trauma scale in a reality which is highly mutable and uncertain. Therefore, future research is needed to look at the reliability and validity of the scale over time and examine the predictive validity of the scale for identifying context-specific trauma exposure using qualitative interviews to establish the validity of the scale as an assessment tool.

## Conclusion

The current study aimed to develop and validate a context-specific trauma checklist to detect PTEs in the Palestinian context. Our research findings showed three stable constructs of TCL in assessing exposure to traumatic events in the Palestinian context. Developing and validating new tools to assess traumatic events in a context characterized by high trauma exposure is crucial in enhancing clinical interventions targeting Palestinian children at risk of political trauma. Life of Palestinian people dramatically deteriorated since the Israeli army's reinvasions of the West Bank in September 2000. Yet, adequate tools for assessing exposure to traumatic events that can inform mental healthcare providers to evaluate and diagnose exposure to PTEs among Palestinian children have been lacking. Interpreting traumatic events with proper and accurate tools will enhance mental health services provided to individuals suffering from ongoing war traumas. This opportunity will also enable healthcare providers to follow and evaluate changes in these cases over different periods. In fact, a robust and easy-to-administer instrument capable of detecting children's exposure to traumatic events is crucial for healthcare providers working in contexts of war and violence. Such an instrument assesses the traumatic experiences to which children are exposed, aiding clinicians in orienting their therapeutic work towards empowering resilience, coping strategies, and survival skills in children. By measuring children's war trauma, psychologists, counselors, social workers, and educators can develop ad hoc participatory interventions tailored to the needs arising during and after armed conflicts. War-related traumas and those associated with civilian-related events (e.g., child abuse, community violence, school bullying), may cause serious mental health problems [29, 30]. Therefore, developing validated assessment tools to assesses war trauma will help healthcare providers establish several therapeutic interventions to prevent PTSD and health outcomes related to PTSD.

## Data Availability

The datasets used and/or analyzed during the current study available from the corresponding author on reasonable request.

## References

[1] Mahamid F, Veronese G (2021). Psychosocial interventions for third-generation Palestinian refugee children: Current challenges and hope for the future. Int J Ment Heal Addict.

[2] UNICEF (2005). State of the World's children 2005.

[3] Mahamid FA (2020). Collective Trauma, Quality of Life and Resilience in Narratives of Third Generation Palestinian Refugee Children. Child Indic Res.

[4] Punamäki RL, Qouta S, El-Sarraj E (2001). Resiliency factors predicting psychological adjustment after political violence among Palestinian children. Int J Behav Dev.

[5] Espié E, Gaboulaud V, Baubet T, Casas G, Mouchenik Y, Yun O, Moro MR (2009). Trauma-related psychological disorders among Palestinian children and adults in Gaza and West Bank, 2005–2008. Int J Ment Health Syst.

[6] Agbaria N, Petzold S, Deckert A, Henschke N, Veronese G, Dambach P, Winkler V (2021). Prevalence of post-traumatic stress disorder among Palestinian children and adolescents exposed to political violence: a systematic review and meta-analysis. PLoS one.

[7] Giacaman R, Mataria A, Nguyen-Gillham V, Safieh RA, Stefanini A, Chatterji S (2007). Quality of life in the Palestinian context: an inquiry in war-like conditions. Health Policy.

[8] Hawkins SS, Radcliffe J (2006). Current measures of PTSD for children and adolescents. J Pediatr Psychol.

[9] Stamm BH, Friedman MJ (2000). International handbook of human response to trauma. Cultural diversity in the appraisal and expression of trauma.

[10] Wilde L (2020). Trauma across cultures: cultural dimensions of the phenomenology of post-traumatic experiences. Phenomenology Mind.

[11] Viola TW, Salum GA, Kluwe-Schiavon B, Sanvicente-Vieira B, Levandowski ML, Grassi-Oliveira R (2016). The influence of geographical and economic factors in estimates of childhood abuse and neglect using the childhood trauma questionnaire: a worldwide meta-regression analysis. Child Abuse Negl.

[12] Choi KR, McCreary M, Ford JD, RahmanianKoushkaki S, Kenan KN, Zima BT (2019). Validation of the traumatic events screening inventory for ACEs. Pediatrics.

[13] Williamson DE, Birmaher B, Ryan ND, Shiffrin TP, Lusky JA, Protopapa J, Brent DA (2003). The stressful life events schedule for children and adolescents: Development and validation. Psychiatry Res.

[14] Elhai JD, Gray MJ, Kashdan TB, Franklin CL (2005). Which instruments are most commonly used to assess traumatic event exposure and post-traumatic effects?: A survey of traumatic stress professionals. J Trauma Stress.

[15] Miller JK, Brewin CR, Soffia M, Elliott-Davies M, Burchell BJ, Peart A (2022). The Development of a UK police traumatic events checklist. Police J.

[16] Pieloch KA, McCullough MB, Marks AK (2016). Resilience of children with refugee statuses: a research review. Can Psychol.

[17] Punamäki RL, Qouta S, El Sarraj E (1997). Models of traumatic experiences and children's psychological adjustment: the roles of perceived parenting and the children's own resources and activity. Child Dev.

[18] Thabet AAM, Vostanis P (1999). Post-traumatic stress reactions in children of war. J Child Psychol Psychiatry.

[19] Thabet AAM, Vostanis P (2014). Impact of Trauma on Palestinian Childrens and the Role of Coping Strategies. British J Med Medical Res.

[20] El-Khodary B, Samara M, Askew C (2020). Traumatic events and PTSD among Palestinian children and adolescents: the effect of demographic and socioeconomic factors. Front Psych.

[21] Thabet AAM, Karim K, Vostanis P (2006). Trauma exposure in pre-school children in a war zone. Br J Psychiatry.

[22] Veronese G, Pepe A (2013). Psychometric Properties of IES-R, Short Arabic Version in Contexts of Military Violence. Res Soc Work Pract.

[23] Veronese G, Fiore F, Castiglioni M, el Kawaja H, Said M (2013). Can sense of coherence moderate traumatic reactions? A cross-sectional study of Palestinian helpers operating in war contexts. Br J Soc Work.

[24] Dyregrov A, Kuterovac G, Barath A (1996). Factor analysis of the impact of event scale with children in war. Scand J Psychol.

[25] Giraldo LS, Aguirre-Acevedo DC, Trujillo S, Ugarriza JE, Trujillo N (2020). Validation of the Extreme Experiences Scale (EX2) for armed conflict contexts. Psychiatr Q.

[26] Ibrahim H, Ertl V, Catani C, Ismail AA, Neuner F (2018). The validity of Post-traumatic Stress Disorder Checklist for DSM-5 (PCL-5) as screening instrument with Kurdish and Arab displaced populations living in the Kurdistan region of Iraq. BMC Psychiatry.

[27] Rabaia Y, Saleh MF, Giacaman R (2014). Sick or sad? Supporting Palestinian children living in conditions of chronic political violence. Child Soc.

[28] Massad S, Khammash U, Shute R (2017). Political violence and mental health of Bedouin children in the West Bank, Palestine: a cross-sectional study. Med Confl Surviv.

[29] Abudayya A, Bruaset GTF, Nyhus HB, Aburukba R, Tofthagen R. Consequences of war-related traumatic stress among Palestinian young people in the Gaza strip: A scoping review. Mental Health Prevention. 2023;23.‏ 10.1016/j.mhp.2023.200305 .

[30] Margolin G, Gordis EB (2000). The effects of family and community violence on children. Annu Rev Psychol.

